# Protease Inhibitor Anti-HIV, Lopinavir, Impairs Placental Endocrine Function

**DOI:** 10.3390/ijms22020683

**Published:** 2021-01-12

**Authors:** Camille Fraichard, Fidéline Bonnet-Serrano, Christelle Laguillier-Morizot, Marylise Hebert-Schuster, René Lai-Kuen, Jeanne Sibiude, Thierry Fournier, Marie Cohen, Jean Guibourdenche

**Affiliations:** 1 INSERM UMR-S 1139, Faculté de Pharmacie, Université de Paris, 75006 Paris, France; camille.fraichard@gmail.com (C.F.); christelle.laguillier@aphp.fr (C.L.-M.); thierry.fournier@parisdescartes.fr (T.F.); 2 Service d’Hormonologie, CHU Cochin, HUPC, AP-HP, 75014 Paris, France; fideline.bonnet@aphp.fr; 3 Service de Gynécologie-Obstétrique, Faculté de Médecine, Université de Genève, 1206 Genève, Suisse; maryliseschuster@gmail.com (M.H.-S.); marie.cohen@hcuge.ch (M.C.); 4 INSERM UMS 025—CNRS UMS 3612, Faculté de Pharmacie, Université de Paris, 75006 Paris, France; rene.lai-kuen@parisdescartes.fr; 5 Service de Gynécologie-Obstétrique, CHU Louis Mourier, HUPN, AP-HP, 92700 Colombes, France; jeanne.sibiude@aphp.fr

**Keywords:** human placenta, lopinavir, progesterone, mitochondria, Mfn2, UPR, IRE1α

## Abstract

Protease Inhibitors (PI e.g., ritonavir (RTV) and lopinavir (LPV)) used to treat pregnant mothers infected by HIV induce prematurity and endocrine dysfunctions. The maintenance of pregnancy relies on placental hormone production (human Chorionic Gonadotrophin (hCG) and progesterone (P4)). Those functions are ensured by the villous trophoblast and are mainly regulated by the Unfolded Protein Response (UPR) pathway and mitochondria. We investigated, in vitro, if PI impair hCG and P4 production and the potential intracellular mechanisms involved. Term villous cytotrophoblast (VCT) were cultured with or without RTV or LPV from 6 to 48 h. VCT differentiation into syncytiotrophoblast (ST) was followed measuring hCG and P4 secretion. We evaluated the expression of P4 synthesis partners (Metastatic Lymph Node 64 (MLN64), cholesterol side-chain cleavage (P450SCC), Hydroxy-delta-5-Steroid Dehydrogenase and 3 Beta-and steroid delta-isomerase 1 (HSD3B1)), of mitochondrial pro-fusion factors (Mitofusin 2 (Mfn2), Optic Atrophy 1 (OPA1)) and of UPR factors (Glucose-Regulated Protein 78 (GRP78), Activating Transcription Factor 4 (ATF4), Activating Transcription Factor 6 (ATF6), spliced X-box Binding Protein 1 (sXBP1)). RTV had no significant effect on hCG and P4 secretion, whereas lopinavir significantly decreased both secretions. LPV also decreased P450SCC and HSD3B1 expression, whereas it increased Mfn2, GRP78 and sXBP1 expression in ST. RTV has no effect on the endocrine placenta. LPV impairs both villous trophoblast differentiation and P4 production. It is likely to act via mitochondrial fusion and UPR pathway activation. These trophoblastic alterations may end in decreased P4 levels in maternal circulation, inducing prematurity.

## 1. Introduction

To prevent mother-to-fetus transmission of Human Immunodeficiency Virus (HIV), World Organization of Health (WHO) recommends to continue or initiate antiretroviral therapy (ART) during pregnancy regardless of the clinical stage or CD4 cell count. ART consists in the association of two NRTI (Nucleoside Reverse Transcriptase Inhibitor) with a Protease Inhibitor (PI) such as Lopinavir (LPV) or Ritonavir (RTV) [1]. However, the use of PI during pregnancy increases the risk of preterm birth and obstetric complications (e.g., pre-eclampsia, diabetes or intra uterine growth restriction) [2,3,4]. Those treatments have been shown to alter both adrenal and placental steroidogenesis. Indeed, neonates exposed in utero to PI exhibit adrenal dysfunction with an increase in 17-OH progesterone [5]. Anti-HIV treatment during pregnancy also induces a decrease in maternal serum progesterone (P4), especially when using PI [6].

From the end of the first month of pregnancy, P4 hormone, like human chorionic gonadotrophin (hCG), is produced by the villous trophoblast in the chorionic villi, mainly the syncytiotrophoblast (ST) [7,8]. We recently showed that the villous cytotrophoblast (VCT), which will differentiate into ST, is also able to produce placental glycoproteic hormones such as hCG and steroids such as P4 in vitro [7,9,10,11]. In placenta, P4 is synthesized from maternal serum cholesterol, which is captured by the trophoblast and enters the mitochondria via Metastatic Lymph Node 64 (MLN64) protein. The cholesterol molecule is then converted by cholesterol side-chain cleavage (P450SCC) enzyme in pregnenolone (P5), which is further converted in P4 by Hydroxy-delta-5-Steroid Dehydrogenase and 3 Beta-and steroid delta-isomerase 1 (HSD3B1) [8,12]. 

The production of hCG and P4 relies on the good functionality of the trophoblast. This functionality is regulated by numerous factors including cyclic Adenosine MonoPhosphate (cAMP)/Protein Kinase A (PKA) pathway, oxidative stress and stress of the Endoplasmic Reticulum (ER). ER stress involves different organelles (mitochondria, ER) and pathways such as the Unfolded Protein Response (UPR) pathway [13,14,15,16,17,18]. 

Mitochondria are essential to ensure energy regulation and steroid hormones production. We previously confirmed a change in mitochondrial function associated with structural modifications during VCT differentiation [11]. Different studies demonstrated that the structural modifications observed between VCT and ST mitochondria are related to mitochondria dynamics, relying on fusion/fission process [19,20,21,22,23]. The fusion is regulated by different factors such as Mitofusin 2 (Mfn2) and Optic Atrophy 1 (OPA1) [24,25,26,27]. In the placenta, mitochondrial dynamics are known to change with trophoblast differentiation but the mechanisms and factors involved remain controversial [18,27,28]. Any disruption in the fusion/fission process may lead to mitochondrial dysfunction, particularly steroidogenesis alteration [29,30].

The ER and Golgi apparatus are key organelles involved in the production of peptide hormones such as hCG [31]. In case of ER stress, an accumulation of unfolded proteins in ER lumen is observed. In response, Glucose-Regulated Protein 78 (GRP78) dissociates from the ER membrane activating the UPR pathway. This pathway involves (i) Inositol-Requiring Enzyme 1α (IRE1α), which induces the splice of X-box Binding Protein 1(XBP1) transcription factor controlling the expression of IRE1α target genes; (ii) Activating Transcription Factor 6 (ATF6), which is cleaved in its active form to control the transcription of its target genes; and (iii) Protein kinase RNA-like ER protein Kinase (PERK), which induces activation of Activating Transcription Factor 4 (ATF4) transcription factor to control the expression of PERK target genes [32]. These UPR pathways are known to regulate trophoblast differentiation, hCG secretion and the steroidogenesis [17,33].

It has been established that anti-HIV treatment by PI alters adrenal steroidogenesis both in the mothers and in their neonates exposed in utero [5,6,34]. As little is known about the effect of PI on the human placenta, we aimed to investigate the effect of two widely used PI (RTV and LPV) on the villous trophoblast differentiation in vitro, its endocrine function, and to identify their potential targets focusing on the mitochondria and the UPR pathway.

## 2. Results

### 2.1. Effect of RTV on the Villous Trophoblast

**Figure 1:**
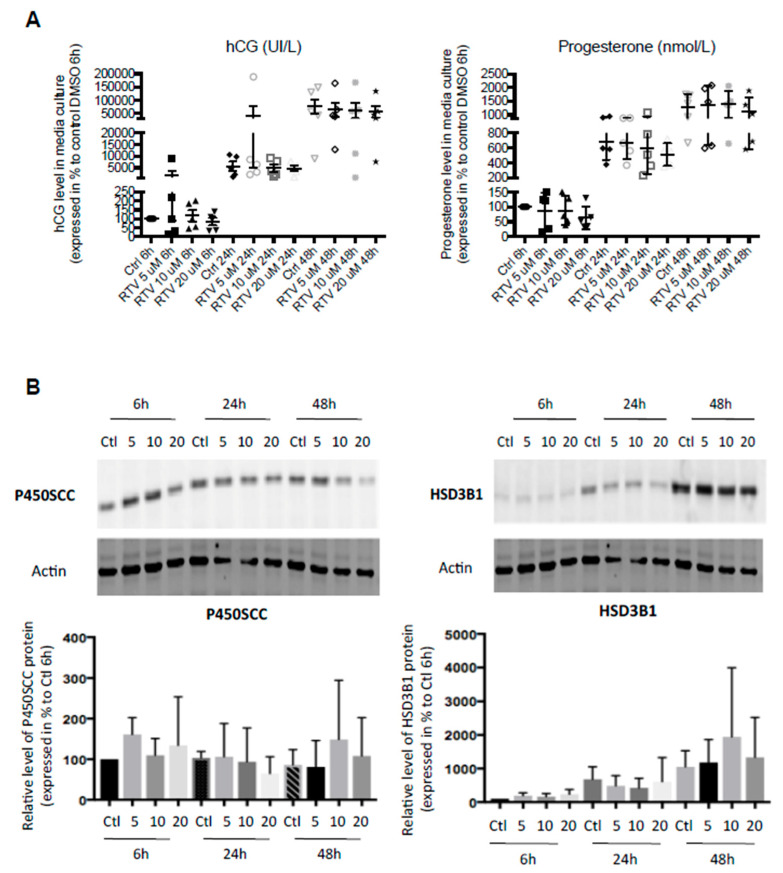
Ritonavir has no effect on hCG and P4 production during trophoblast differentiation. Cytotrophoblasts isolated and purified from term placenta were cultured for 15 h and then incubated with RTV at 5, 10 or 20 µM or DMSO for 6 h, 24 h or 48 h to allow fusion process. (**A**) hCG concentration and P4 concentration were measured by immuno-analysis in culture supernatant. (**B**) The protein expression of P450SCC and HSD3B1 were evaluated using immunoblotting with anti-P450SCC and anti-HSD3B1 antibodies. The protein expression of actin was determined with anti-actin antibody, used as a loading control. The lanes intensity was measured with ImageJ program. Results are expressed as the mean +/− SEM of *n* = 6 independent experiments. Two-tailed paired no parametric student t-tests were performed to compare RTV to DMSO exposition at the same incubation time.

hCG and P4 levels were measured in supernatants of trophoblast cells incubated with RTV or control dimethylsulfoxide (DMSO). Neither hCG nor P4 secretion was disrupted during differentiation of VCT into ST whatever the incubation time (6 to 48 h) or RTV concentration (5 to 20 µM) *(*Figure 1A). On Western blot, the expression of P450SCC and HSD3B1, two enzymes involved in P4 synthesis, was not affected during RTV exposition (Figure 1B). RTV had no effect on villous trophoblast differentiation (data not shown).

### 2.2. Effect of LPV on the Villous Trophoblast

**Figure 2:**
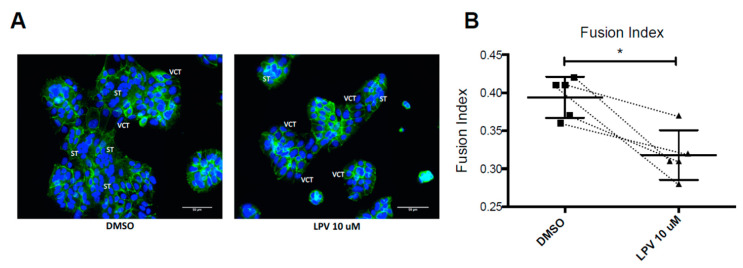
Lopinavir decreases VCTs fusion. VCT isolated and purified from term placenta were cultured for 15 h and then incubated with LPV 10 µM or DMSO for 48 h to allow fusion process. (**A**) Picture of cytotrophoblast fusion process by fixing and immunostaining of cells for the distribution of desmoplakin (green) and nuclei (4′,6-diamidino-2-phenylindole [DAPI] staining). 400 × magnification. Scale bar: 50 µm. (**B**) Representation of syncytium formation as a fusion index graph. Results are expressed as the mean +/− SD of *n* = 5 independent experiments. * *p* < 0.05 vs. DMSO, Mann-Whitney *t*-test.

In controls, VCT spontaneously fuses to form a ST at 72 h of culture. After 48 h of incubation with LPV at 10 µM, ST formation was decreased as demonstrated with desmoplakin staining distribution (Figure 2A). The fusion index calculation points out a significant decrease (*p* < 0.05) of 20% in VCT fusion into ST after 48 h of incubation with LPV at 10 µM (Figure 2B). 

**Figure 3:**
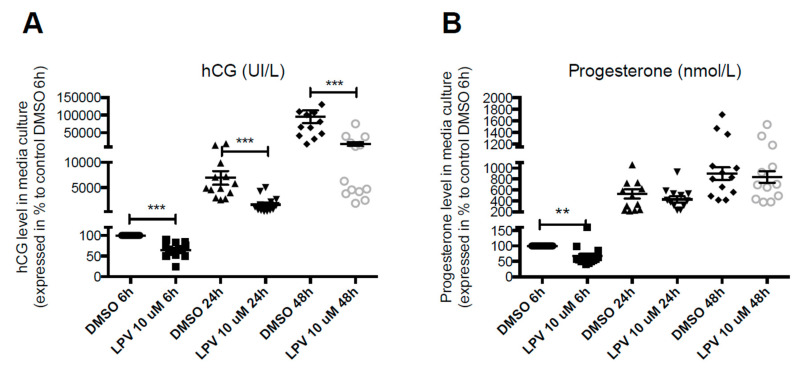
Lopinavir decreases hCG and progesterone secretion during differentiation of VCT into ST. Cytotrophoblasts isolated and purified from term placenta were cultured for 15 h and then incubated with LPV 10 µM or DMSO for 6 h, 24 h or 48 h to allow fusion process. hCG concentrations (**A**) and progesterone concentrations (**B**) were measured by immuno-analysis in culture supernatant. Results are expressed as the mean +/− SEM of *n* = 11 independent experiments. ** *p* < 0.01; *** *p* < 0.001 vs. DMSO at the same incubation time, two-tailed paired no parametric student *t*-test.

As expected, VCT fusion into ST was associated with an increase in hCG (by 1,000-fold) and P4 (by 10-fold) secretion in controls. LPV at 10 µM significantly (*p* < 0.001) decreased hCG secretion by 35% in average after 6 h of incubation, reaching 84% of decrease at 48 h of incubation (Figure 3A). LPV also induced an early significant (*p* < 0.01) decrease in P4 secretion by 41% in average that tended to disappear thereafter (Figure 3B). 

### 2.3. Expression of Trophoblastic Enzymes Involved in P4 Synthesis during LPV Exposition

**Figure 4:**
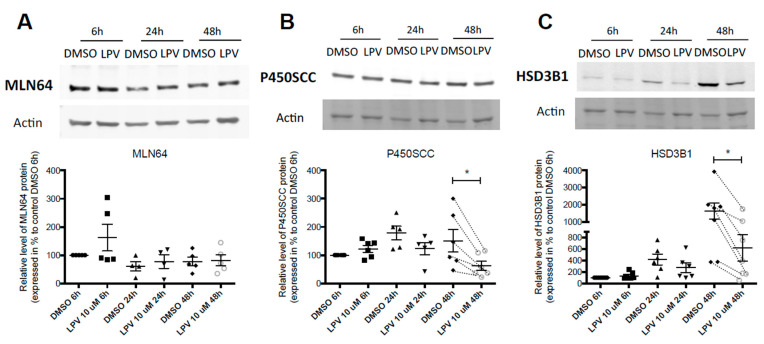
Lopinavir decreases protein expression of enzymes P450SCC and HSD3B1 involved in progesterone synthesis during differentiation of VCT into ST. Human VCT isolated and purified from term placenta were cultured for 15 h and then incubated with LPV 10 µM or DMSO for 6 h, 24 h or 48 h. MLN64 (**A**), P450SCC (**B**) and HSD3B1 (**C**) protein expression was determined using immunoblotting with anti-MLN64, anti-P450SCC and anti-HSD3B1 antibodies. Actin protein was determined with anti-actin antibody, used as a loading control. The lanes intensity was measured with ImageJ program. Results are expressed as a percentage of the control DMSO 6 h conditions and are shown as mean −/+ SEM from six independent experiments. * *p* < 0.05 vs. DMSO at the same incubation time, two-tailed paired no parametric paired *t*-test.

As only LPV decreases both ST formation and P4 secretion, P4 synthesis partners were further investigated. On Western blots, LPV significantly (*p* < 0.05) decreased expression of P450SCC enzyme by 58% in average and HSD3B1 enzyme by 62% in average after 48 h of incubation (Figure 4B,C). However, LPV did not affect expression of mitochondrial cholesterol transporter MLN64 whatever the incubation time (Figure 4A). 

### 2.4. Trophoblastic Nuclei, Mitochondria and Endoplasmic Reticulum under LPV Treatment

**Figure 5:**
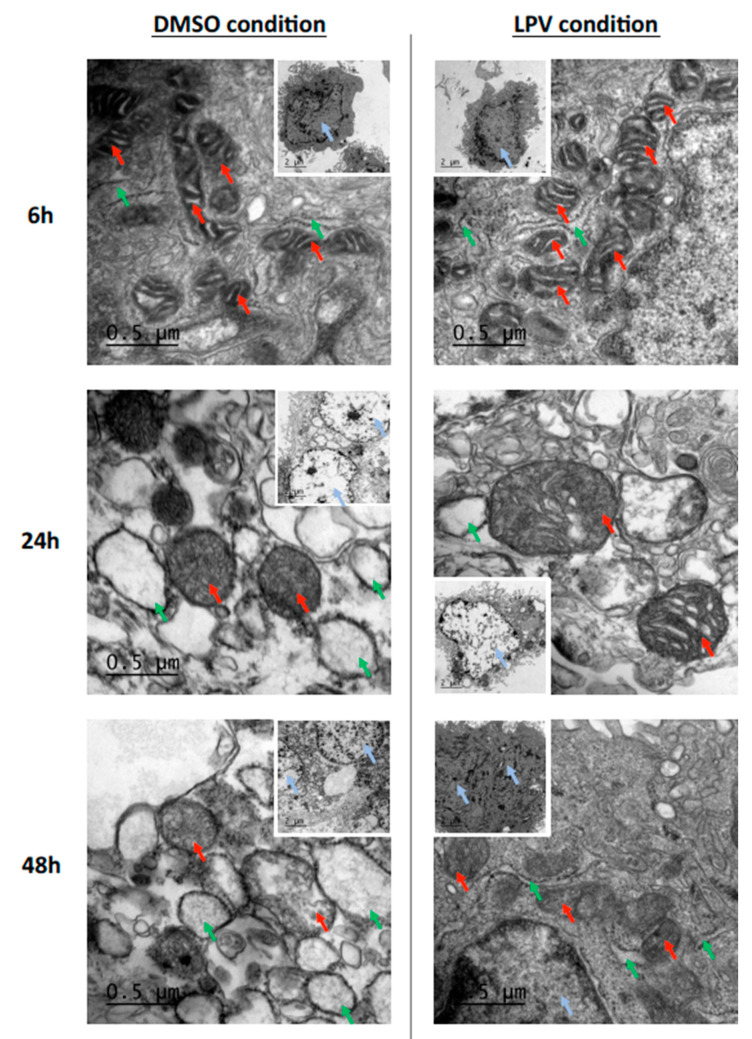
Lopinavir alters mitochondria and endoplasmic reticulum structure during trophoblast differentiation. Cytotrophoblasts isolated and purified from term placenta were cultured for 15 h and then incubated with LPV 10 µM or DMSO for 6 h, 24 h or 48 h. Cells have been fixed and prepared for transmission electronic microscopy. The blue, red and green arrows indicate respectively the nuclei, mitochondria and endoplasmic reticulum (ER). 1000× magnification was used for the small pictures; scale bar: 2 µm. Zoom in 5000× magnification was used for the principal pictures. Scale bar: 0.5 µm. These pictures are representative of 3 independent experiments.

We then analyzed by electron microscopy two main organelles involved in P4 and hCG synthesis, respectively. In controls, at 6 h, i.e., when VCT are still predominant and ST not yet formed, mitochondria present a few dense matrix with clearly defined cristae (Figure 5). On the contrary, at 48 h, i.e., when the vast majority of VCT has differentiated into ST, mitochondria present a clearly denser matrix with a less defined and more atypical cristae structure than in VCT (Figure 5). Moreover, we observed an increase in nuclei chromatin condensation with differentiation of VCT into ST (Figure 5). In VCT cells (i.e., 6 h of incubation), ER is thin, while in ST (i.e., 48 h of incubation), ER is larger (Figure 5). These physiological changes were not modified under LPV treatment in VCT (6 h). On the contrary, in ST (48 h), chromatin was less condensed than in controls and ER was thinner and rather empty. Mitochondria presented a less dense matrix with clearly defined cristae (Figure 5). In cells incubated with LPV for 24 h, the results were less significant as some VCT had not already started their differentiation.

### 2.5. Mitochondrial Dynamics in Villous Trophoblast under LPV

**Figure 6:**
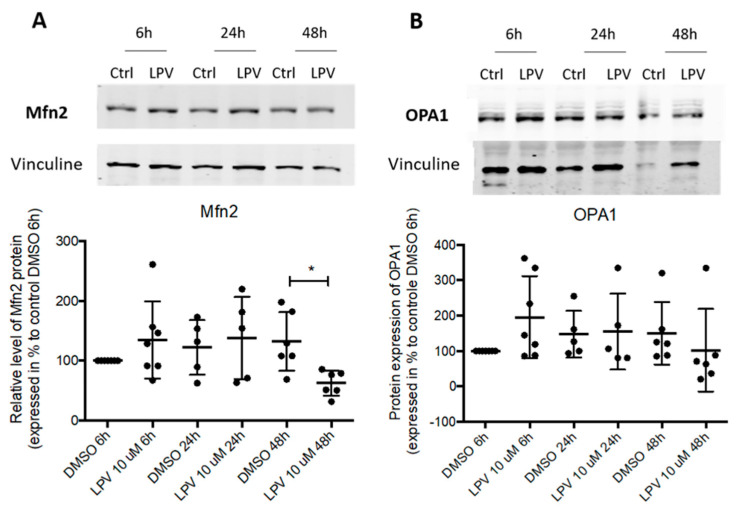
Impairment in mitochondrial dynamics during trophoblast differentiation under LPV exposition. Cytotrophoblast cells isolated from human term placentas were cultured for 15 h before to the incubation with LPV 10 µM or DMSO for 6 h, 24 h or 48 h. Mfn2 (**A**) or OPA1 (**B**) protein expression were evaluated by immunoblotting with anti-Mfn2 and anti-OPA1 antibodies. Vinculin protein expression determined with anti-vinculin antibody was used as loading control. Results are expressed as the mean +/− SD of *n* = 7 independent experiments. * *p* < 0.05 vs. DMSO at the same incubation time, two-tailed paired no parametric student *t*-test.

The mitochondrial structure relies on the fusion and fission process regulated by several proteins (OPA1, Mfn2). LPV induced a significant decrease (*p* < 0.05) in Mfn2 protein expression after 48 h incubation (Figure 6A), while OPA1 protein expression was not affected (Figure 6B).

### 2.6. ER Stress in Villous Trophoblast under LPV

**Figure 7:**
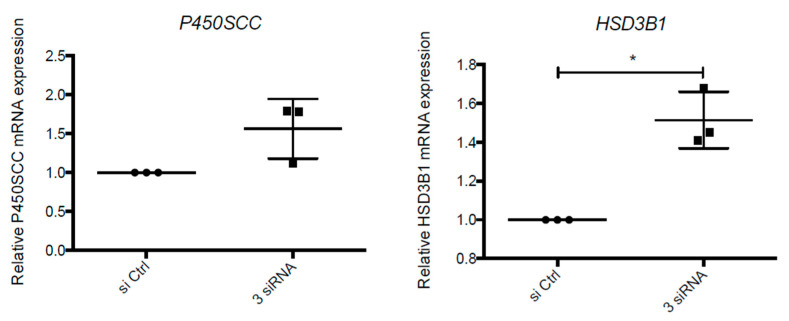
UPR pathways are involved in transcriptional regulation of enzymes involved in P4 synthesis. VCT cells isolated from human term placenta were transfected with siRNA against *IRE1α*, *PERK* and *ATF6* (3 siRNA) or siRNA control (si Ctrl). Total RNA was extracted and *P450SCC* and *HSD3B1* expression were evaluated by RT-qPCR. The results are presented as mean +/− SD of 3 independent experiments. * *p* < 0.05 vs. si Ctrl, two-tailed paired no parametric *t*-test.

In collaboration with Dr Marie Cohen, we first checked whether UPR pathways are involved in steroidogenesis regulation in our in vitro model. We evaluated mRNA expression of *P450SCC* and *HSD3B1* by RT-qPCR on VCT cells transfected with 3 siRNA against *IRE1α*, *PERK* and *ATF6*, respectively. Inhibition of UPR pathway tended to induce an increase in *HSD3B1* and *P450SCC* expression in the same proportion (50% and 55%, respectively) (Figure 7). 

**Figure 8:**
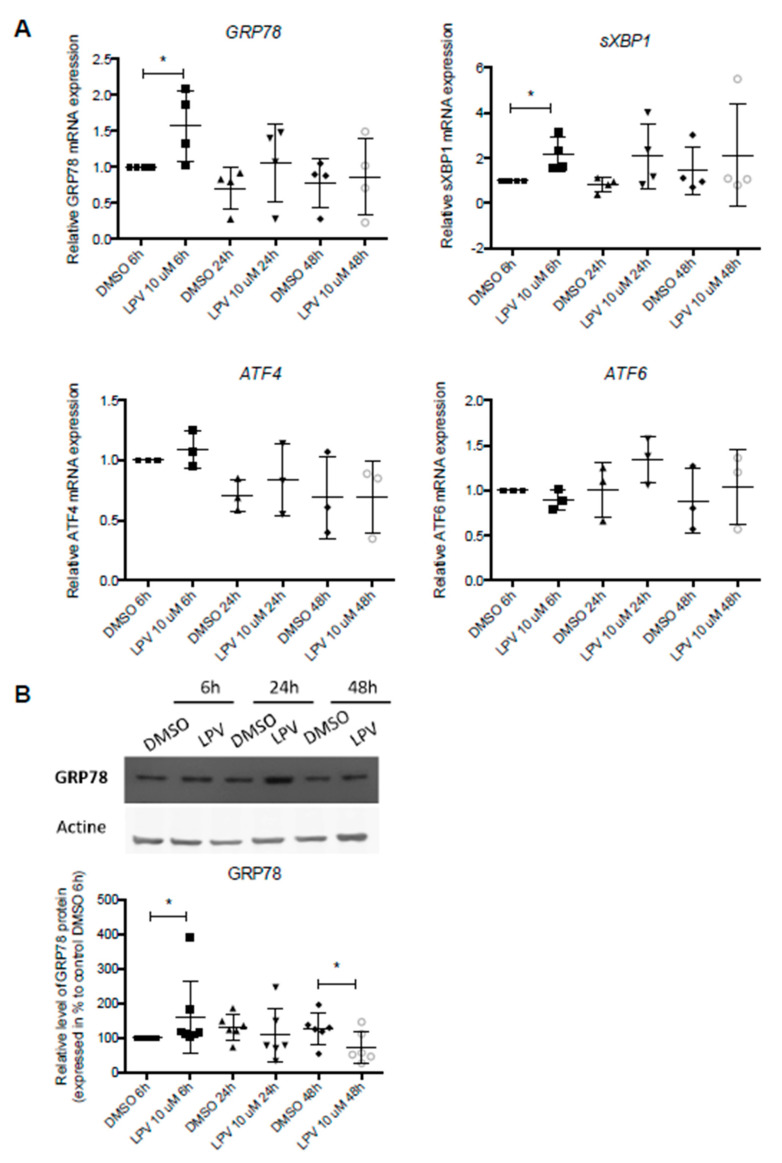
LPV induces activation of IRE1α pathway from 6 h of incubation in trophoblasts cells. VCT cells were isolated from human term placenta. After 15 h of culture, cells were incubated for 6 h, 24 h or 48 h with 10 µM LPV. (**A**) Transcriptional expression of *GRP78*, *sXBP1*, *AFT4* and *AFT6* were measured by RT-qPCR. (**B**) Protein expression of GRP78 was evaluated by immunoblotting with anti-GRP78 antibody. Actin protein expression determined with anti-actin antibody was used as loading control. The results are expressed as mean +/− SD of *n* = 4 independent experiments. * *p* < 0.05 vs. DMSO at the same incubation time, two-tailed paired no parametric *t*-test.

We then investigated whether LPV activates UPR pathways measuring mRNA expression of UPR markers: *GRP78*, *ATF6*, *ATF4* and *sXBP1* by qPCR. In VCT (i.e., 6 h incubation with LPV), *GRP78* and *sXBP1* expression significantly increased 1.5- and 2-fold (*p* < 0.05) (Figure 8A). During trophoblast differentiation, GRP78 protein expression under LPV increased 1.5-fold in VCT cells, after 6 h of incubation but decreased 0.65-fold in ST, after 48 h of incubation (Figure 8B).

### 2.7. Effects of LPV on Preformed ST

**Figure 9:**
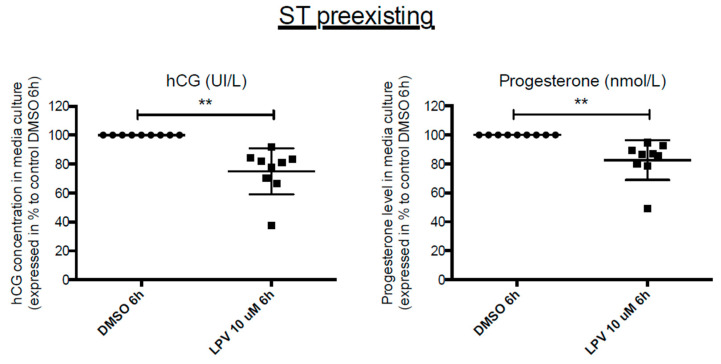
Brief incubation with Lopinavir decreases hCG and progesterone secretion both in VCT and ST cells. Cytotrophoblasts isolated and purified from term placenta were cultured for 72 h in complete DMEM to allow ST formation before incubation for 6 h with LPV 10 µM or DMSO. hCG and progesterone concentrations were measured by immuno-analysis in culture supernatant. Results are expressed as the mean +/− SEM of *n* = 11 independent experiments. ** *p* < 0.01 vs. DMSO, two-tailed paired no parametric student *t*-test.

As ST is trophoblastic tissue in direct contact with maternal blood, we analyzed the impact of LPV directly on ST (i.e., when VCT differentiation is already performed). VCT were cultured in complete Dulbecco’s Modified Eagle Medium (DMEM) for 72 h forming the ST. The resulting ST was further exposed to LPV at 10 µM for 6 h. Exposition to LPV induced a significant decrease in hCG and P4 secretion by 20% and 40%, respectively (*p* < 0.05) (Figure 9).

**Figure 10:**
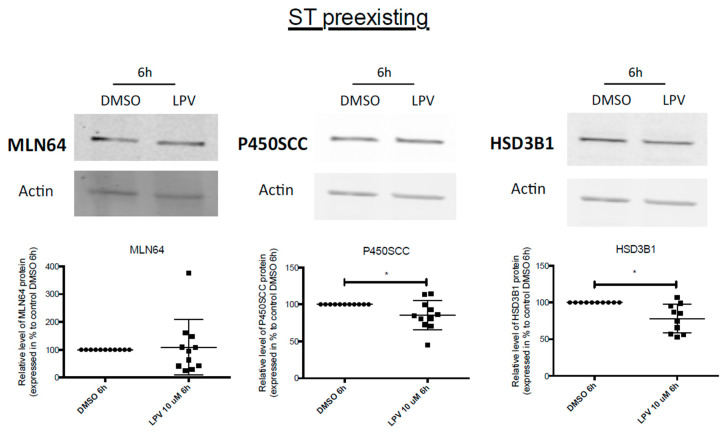
Decrease in enzymes protein expression in ST cells under brief LPV exposition. Cytotrophoblast cells isolated from human term placentas were cultured for 72 h before incubation with LPV 10 µM or DMSO for 6 h. MLN64, P450SCC and HSD3B1 protein expression was evaluated by immunoblotting with anti-MLN64, anti-P450SCC and anti-HSD3B1 antibodies. Actin protein expression determined with anti-actin antibody was used as loading control. Results are expressed as the mean +/− SD of *n* = 8 independent experiments. * *p* < 0.05 vs. DMSO, two-tailed paired no parametric student *t*-test.

P450SCC and HSD3B1 protein expression was also significantly decreased by 20% in ST exposed to LPV for 6 h (*p* < 0.05) (Figure 10).

**Figure 11:**
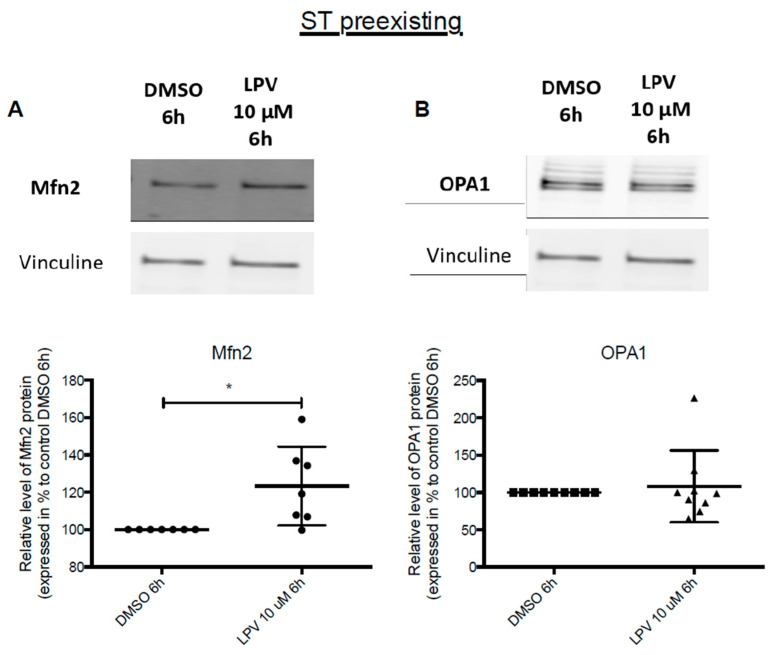
Impairment in mitochondrial dynamics in ST cells under LPV exposition. Cytotrophoblast cells isolated from human term placentas were cultured for 72 h before incubation with LPV 10 µM or DMSO for 6 h. Mfn2 (**A**) and OPA1 (**B**) protein expression was evaluated by immunoblotting with anti-Mfn2 and anti-OPA1 antibodies. Vinculin protein expression determined with anti-vinculin antibody was used as loading control. Results are expressed as the mean +/− SD of *n* = 7 independent experiments. * *p* < 0.05 vs. DMSO, two-tailed paired no parametric student *t*-test.

Exposition of ST to LPV for 6 h also induced a significant increase by 20% in Mfn2 mitochondrial protein expression (*p* < 0.05) (Figure 11A), while OPA1 mitochondrial protein expression was unchanged (Figure 11B). 

**Figure 12:**
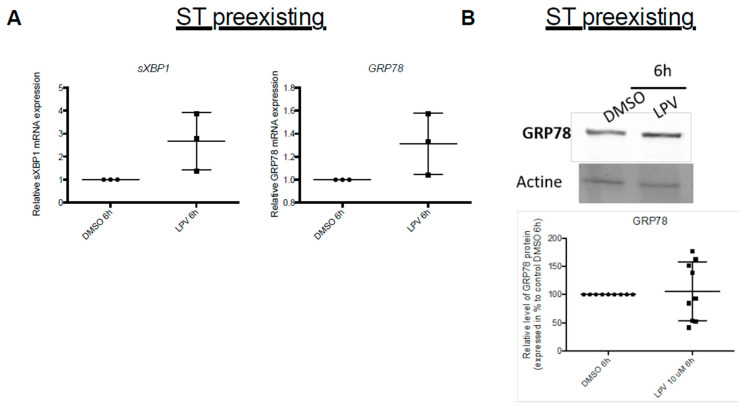
Activation of UPR pathway in VCT cells under LPV exposition. Cytotrophoblast cells isolated from human term placentas were cultured for 72 h before incubation with LPV 10 µM or DMSO for 6 h. (**A**) mRNA expression of *sXBP1* and *GRP78* were measured by RT-qPCR. The results are expressed as mean +/− SD of *n* = 3 independent experiments. Two-tailed paired no parametric *t*-test were realized. (**B**) GRP78 protein expression was evaluated by immunoblotting with anti-GRP78 antibody. Actin protein determined with anti-actin antibody was used as loading control. The results are expressed as mean +/− SD of *n* = 6 independent experiments. Two-tailed paired no parametric *t*-test were realized.

Exposition of ST to LPV tended to activate UPR pathway as attested by the increase in *GRP78* and *sXBP1* transcripts (Figure 12A).

**Figure 13:**
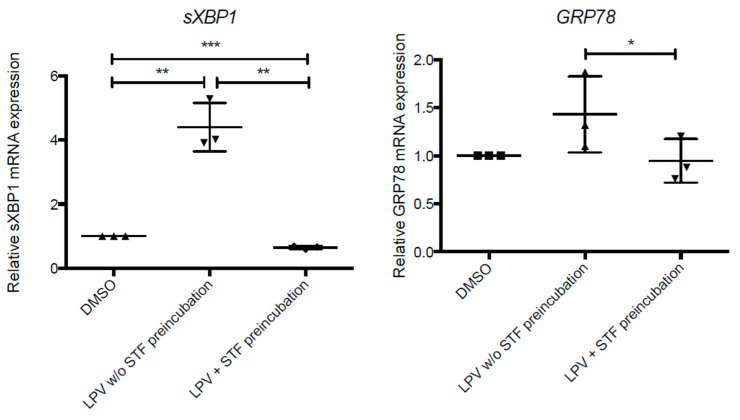
Inhibition of IRE1α pathway partially prevents the UPR pathway activation. Cytotrophoblast cells isolated from human term placentas were cultured for 72 h. Cells were then incubated for 2 h with STF (100 µM) (LPV with STF preincubation) or DMSO control (LPV without (*w/o*) STF preincubation) before incubation for 4 h with LPV (10 µM). (A) mRNA expression of *sXBP1* and *GRP78* were measured by RT-qPCR. The results are expressed as mean +/− SD of *n* = 3 independent experiments. * *p* < 0.05; ** *p* < 0.01; *** *p* < 0.001 vs. DMSO, two-tailed paired no parametric *t*-test.

We investigated whether the IRE1α-pathway was targeted by LPV. Using STF-083010 (STF), an IRE1α inhibitor, we checked that IRE1α inhibition led to a decrease in LPV effects on UPR pathway, quantifying *sXBP1* and *GRP78* gene expression by RT-qPCR (Figure 13). 

**Figure 14:**
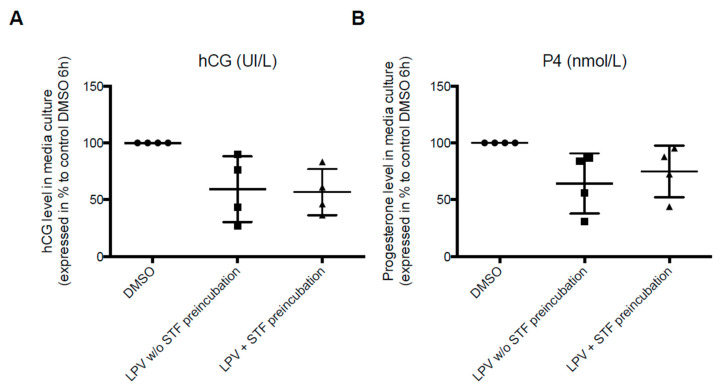
Inhibition of IRE1α pathway does not prevent the inhibitory effect of LPV on hCG and P4 secretions. VCT cells isolated from human term placenta were cultured for 72 h. Cells were further incubated for 2 h with STF (100 µM) (LPV + STF preincubation) or DMSO control (LPV without (*w/o*) STF preincubation) before incubation for 4 h with LPV (10 µM). hCG (**A**) and P4 (**B**) levels were measured in supernatant. The results are presented as the mean +/− SD from four independent experiments.

We then evaluated the effect of IRE1α inhibition on hCG and P4 production by ST under LPV exposition. Pre-incubation with STF-083010 (STF), the IRE1α inhibitor, did not restore hCG and P4 secretion, decreased by LPV in ST (Figure 14). 

**Figure 15:**
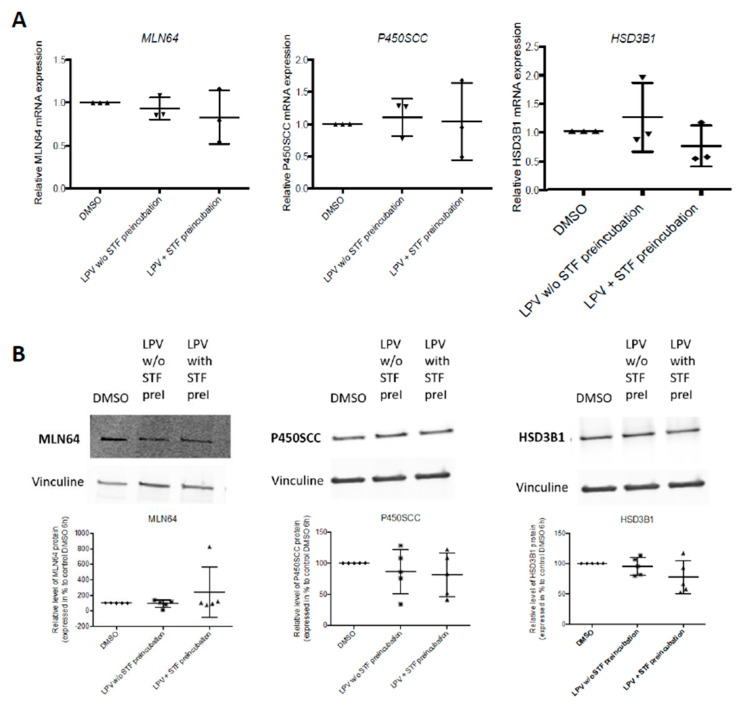
Inhibition of IRE1α pathway does not prevent the inhibitory effect of LPV on P4 synthesis partners expression. VCT cells isolated from human term placenta were cultured for 72 h. Cells were further incubated for 2 h with STF (100 µM) (LPV + STF preincubation) or DMSO control (LPV without (*w/o*) STF preincubation) before incubation for 4 h with LPV (10 µM). MLN64, P450SCC and HSD3B1 expression was evaluated by RT-qPCR (**A**) and Western blot using anti-MLN64, anti-P450SCC and anti-HSD3B1 antibodies (**B**). Vinculin protein expression determined by Western blot using anti-vinculin antibody was used as loading control. The results are presented as the mean +/− SD from *n* = 3 independent experiments for RT-qPCR and *n* = 5 independent experiments for Western blot.

In addition, this pre-incubation did not modify the expression of either P450SCC or HSD3B1 (Figure 15) at both mRNA and protein levels.

## 3. Discussion

During pregnancy, HIV-infected mothers are treated with two NRTI and one PI to prevent the viral transmission to the fetus [1]. According to several studies those treatment, notably the PI, have secondary effects such as pre-term birth, pre-eclampsia, and intra uterine growth restriction [3,4]. Moreover, PI impair steroidogenesis, inducing a decreased P4 level in maternal blood and impaired adrenal function in neonates exposed in utero [3,5,6]. However, very little is known about the placenta, which produces P4, a steroid hormone mandatory for the maintenance of pregnancy [7,8,35]. Our aim was to investigate in vitro whether PI such as RTV and LPV disturb human placental steroidogenesis, focusing on the P4, mitochondria and main intracellular pathways potentially targeted.

For many years, the syncytiotrophoblast (ST) has been considered as the main endocrine tissue of the placenta as it is in direct contact with maternal blood in the intervilli chamber [36]. As in the chorionic villi, the ST arises from the differentiation of the villous cytotrophoblast (VCT), we used our in vitro model to extensively characterize P4 production in human villous placenta. In our previous study [11], we established that placental steroidogenesis is not restricted to ST only but starts early in the VCT, which expresses the cholesterol transporter MLN64 and the key enzymes P450SCC and HSD3B1 required for P4 synthesis and secretes significant levels of P4. We confirmed these findings in this work, pointing out that the whole trophoblast is able to produce placental steroid and protein hormones. This is in agreement with previous studies showing that VCT and also extravillous cytotrophoblast (EVCT) produce significant amounts of hCG and its subunits [37]. We confirmed in this in vitro study that the endocrine production increases with the morphological differentiation of VCT into ST, hCG secretion increase being associated with ST formation. We also established that this increase in hCG secretion is correlated with an increase in P4 synthesis and secretion [11]. 

The mitochondria is a key organelle involved both in steroidogenesis and in trophoblast differentiation [16,27,30]. We have previously demonstrated that the differentiation of VCT into ST is associated with a decrease in mitochondrial transmembrane potential. This functional change in mitochondria could explain the observed changes in P4 synthesis [11]. Indeed, a previous study on placental tissue demonstrated that a decrease in transmembrane potential in mitochondrial fraction is associated with a high steroidogenesis activity [38]. In addition, we showed that in vitro differentiation of VCT into ST is associated with morphological changes in mitochondria using transmission electronic microscopy. Indeed, in our in vitro model, VCT are predominant at 24 h of culture, whereas ST is formed at 72 h of culture. In VCT, mitochondria are larger than in ST. Moreover, mitochondria in VCT present cristae with typical structure, whereas they present in ST an atypical structure of cristae and a denser matrix than in VCT, in agreement with previous histological studies [20]. These structural modifications are known to be related to fusion/fission dynamics of mitochondria [21]. However, in our model, we observed no modification in the expression of two main fusion proteins Mfn2 and OPA1 during differentiation of VCT into ST. These results are not consistent with a previous study in BeWo cells that demonstrated a decrease in Mfn2 and OPA1 expression but, under forskolin, induced differentiation [27]. This suggests that the observed changes in mitochondria depend on the model used, i.e., primary trophoblast culture in our model compared to choriocarcinoma cell lines and on experimental conditions, i.e., basal or stimulated by forskolin. Mitochondrial dynamics are known to be involved in the regulation of steroidogenesis. Some studies demonstrated that fission process is necessary for steroidogenesis [22,27], while others reported on the contrary that steroidogenesis relies on fusion process [30,39]. In our model, it is likely that fusion process is not necessary for P4 synthesis as we found no change in Mfn2 and OPA1 expression during the ST formation. It would thus be of interest to evaluate the expression of other factors such as fission factors Drp1 and Fiss1. An increase in Peroxisome proliferator-activated receptor Gamma Coactivator 1-α (PGC1-α) expression induced by P4 could lead to an increase in mitochondria biogenesis allowing an increase in P4 synthesis itself [40]. 

We also found morphological changes in nuclei and ER during VCT differentiation into ST. In ST, we observed that the nuclei present a denser chromatin compared to VCT, confirming previous placental histological findings [36]. The ER is also thinner in VCT than in ST cells. The presence of large ER in ST is associated with an intense protein synthesis [41,42], consistent with the observed increase in hCG secretion. It is also in agreement with the UPR activation during VCT differentiation into ST [17]. To conclude, the use of transmission electronic microscopy allowed us to fully characterize the differentiation of VCT into ST at cellular level, pointing out a membrane fusion, a nuclear high-condensed chromatin, small and dense mitochondria and large ER. This is in agreement with in vivo findings showing a release of syncytial knots containing ST DNA and UPR activation and an increased hormonal production.

We tested the impact of PI, notably the LPV, at a concentration of 10 µM, which corresponds to maternal blood concentration. Higher concentrations are likely to induce apoptosis as observed in HEK293 cells [43]. LPV at 10 µM induced an early decrease in P4 production in VCT that could be in part responsible for the decrease in P4 level in treated mother serum [3,6]. However, it would be of interest to investigate the effect of LPV/RTV as RTV is often associated to LPV as a booster [3]. Surprisingly, the decrease in P4 secretion under LPV was not associated with a decrease in expression of P450SCC and HSD3B1 enzymes in VCT. LPV could act rather indirectly by modulating P4 catabolism or transport. Indeed, LPV could activate Cytochrome P450 (CYP) family enzymes [44] and could modulate Adenosine-Tri-Phosphate (ATP)-Binding Cassette transporter activity [45]. Moreover, in BeWo cells, Papp et al. have demonstrated that PI could act on 20-α Hydroxysteroid Dehydrogenase, an enzyme involved in progesterone catabolism [46]. Consequently, in short incubation times, PI could decrease progesterone secretion in inhibiting enzymes involved in its synthesis and in activating enzymes involved in its catabolism. In ST, after 48 h of incubation with LPV, we also noted a discrepancy between the decreased expression of P450SCC and HSD3B1 enzymes, and the level of P4 secretion. The decrease in enzyme expression could be due to a global alteration in protein synthesis involving the eucaryotic Elongation Factor-2 (eEF2) translation factor. Indeed, it has previously been shown in the myocyte that LPV induces a decrease in protein synthesis [47]. The absence of P4 decrease after 48 h of incubation with lopinavir, while P450SCC and HSD3B1 expression is reduced, could be explained by the high stability of steroid hormones in our media culture. Consequently, we are not able to detect significant changes in P4 concentration after 48 h of incubation. So, it would be interesting to measure P4 concentration at shorter time or by changing our media culture.

LPV did not only induce an early decrease in P4 but also in hCG secretion in VCT. However, the decrease in hCG also persisted in ST after 48 h of incubation. This could be due to the fact that LPV affects rather ER and protein pathway than mitochondria and steroidogenesis. During ST formation, dynamic changes in some mitochondria could overlap the initial effect of LPV observed on VCT. On the contrary, as far as hormone production is concerned, we observed that LPV leads to a decrease in plasma membrane fusion as demonstrated by the decrease in fusion index associated with a decreased nuclei chromatin condensation and a thinner ER. Consequently, the 48 h incubation with LPV may prevent the morphological changes physiologically associated with functional differentiation. However, it is possible that the early decrease in P4 and hCG secretion induced by LPV from 6 h of incubation in VCT is responsible for the reduced morphological differentiation as both hormones are involved in autocrine an paracrine regulation of the trophoblast differentiation. As a matter of fact, hCG receptors are more expressed in VCT cells than in ST [13] and could be regulated by P4 [48] in order to control plasma membrane fusion [14]. Consequently, the decrease in P4 observed in VCT cells could impair hCG receptor expression. The decrease in hCG receptors expression and hCG secretion would lead to a decrease in plasma membrane fusion, preventing the global differentiation of VCT into ST.

Several intracellular mechanisms are involved in trophoblast differentiation [13,14] such as mitochondria dynamics and UPR pathways [16,17]. It is known that PI, notably LPV, alter both mitochondria DNA content and dynamics [49]. We analyzed the protein expression of two mitochondrial pro-fusion factors Mfn2 and OPA1. We demonstrated that LPV induced a decrease in Mfn2 expression after 48 h of incubation while OPA1 expression remained unchanged. The decrease in at least one fusion factor could result in a decrease in fusion process. These results are consistent with previous studies demonstrating that mitochondrial fusion is necessary for steroidogenesis [30,39]. However, they are inconsistent with the observed P4 secretion in ST after 48 h of incubation. This discrepancy could be explained by a global decrease in protein synthesis including Mfn2. It could be induced by a global cellular stress targeting rather the ER and the proteins than the mitochondria and the steroidogenesis [47].

Marie Cohen et al. also previously demonstrated that trophoblast differentiation and hCG secretion are under the control of UPR pathway [17]. Interestingly, several studies showed an activation of UPR, especially the IRE1α pathways under LPV exposition [43,50,51,52,53]. In collaboration with Dr Marie Cohen, we firstly demonstrated that UPR pathway regulates the expression of enzymes involved in P4 synthesis in placenta. We established in our model that LPV induces an early increase in sXBP1 and GRP78 expression in VCT pointing out an activation of IRE1α pathway in agreement with previous studies [43,50,51,52,53]. The activation of IRE1α is not maintained in ST, where GRP78 expression is on the contrary decreased. All these findings under LPV exposition, i.e., UPR activation, and decrease in GRP78, P450SCC, HSD3B1 and Mfn2, are likely to reflect an uncontrolled global cellular stress. Physiologically, UPR activation allows the maintenance of cellular homeostasis in case of stress. During a stress which cannot be controlled by UPR pathways activation, other processes take place, including particularly a general decrease in protein synthesis [32]. Thus, LPV could induce a stress activating IRE1α pathways. This stress could become uncontrollable after 48 h incubation with LPV ending in a global cell alteration and decreased protein synthesis as previously demonstrated in myocytes cells [47]. In summary, LPV have dual effects, an early effect (after 6 h of incubation) on enzyme and transporter activities to reduce P4 secretion and a later effect (after 48 h of incubation) on protein synthesis due to an ER stress.

In comparison to other PI, we demonstrated that RTV has no effect either on the ST formation or on hCG and P4 secretion in vitro, whatever the used concentration.

LPV exposition could not only disrupt VCT and its functional differentiation into ST, but it could also directly damage the ST covering the villi in direct contact with maternal blood and thus with PI. Indeed, on preexisting ST, we showed that LPV induces a decrease in both hCG and P4 secretion. In ST, on the contrary of VCT, this decreased secretion is associated with a decrease in P450SCC and HSD3B1 expression. LPV induces an increase in Mfn2 expression in ST. Consequently, the mitochondria in ST are more fused under LPV exposition. The increase in mitochondrial fusion induced by LPV could explain the decrease in P4 secretion by ST. We also investigated the effect on UPR pathways. In the preexisting ST, LPV induces an increase in sXBP1 expression demonstrating the activation of IRE1α pathway. To investigate whether this activation is responsible for the disruption in hCG and P4 secretion, we used an IRE1α inhibitor. We showed that the IRE1α inhibitor does not prevent either the decrease in hCG and P4 secretion or the decrease in P450SCC and HSD3B1 expression induced by LPV. The absence of effect of the IRE1α inhibitor may be explained by compensatory mechanisms of other UPR pathways. Indeed, the use of chemical UPR inhibitors is known to lead to compensatory mechanisms via the activation of the other UPR pathways [54]. It will thus be of interest to perform additional experiments combining different chemical inhibitors or siRNA targeting the 3 UPR pathways. Further investigation would also be necessary to check whether LPV directly activates UPR pathways or not. As a matter of fact, in HEK293 cells, Taura et al. [43] demonstrated that LPV is able to produce Reactive Oxygen Species (ROS), leading to an activation of JUNK kinase pathway, further activating UPR pathways. Consequently, ROS production could be involved in the indirect effect of LPV on UPR activation in trophoblast.

The treatment of HIV infection uses a combination of two NRTI combined with a PI. In our study, we have demonstrated for the first time that RTV has no significant effects on endocrine function in trophoblast cells. On the contrary, LPV induces alteration of trophoblast morphological differentiation related to a decrease in plasma membrane fusion and in nuclei chromatin condensation, larger mitochondria and a thinner ER. These morphological alterations are associated with a decrease in P4 and hCG secretions. LPV also impairs mitochondrial dynamics as attested by an increase in Mfn2 expression and an induction of ER stress. ER stress leads to IRE1α activation marked by an increase in sXBP1 and GRP78 expression. These findings give the beginning of an explanation for the PI in vivo toxicity as they are known to induce preterm birth especially when they are “boosted” with RTV.

## 4. Materials and Methods 

### 4.1. Placental Tissue Collection

Placental tissues from patients delivering by cesarean section at full term (mean gestational age: 39 ± 1 weeks of gestation) were obtained from Antoine Béclère Hospital (Clamart, France), Antony Hospital (Antony, France), Port-Royal Maternity (Paris, France) and Montsouris Mutual Institute (Paris, France). Indications for cesarean sections were: maternal uterus abnormalities (uterus scar, myomectomy), maternal wish, breech presentation, narrow maternal pelvis. Placentas were collected following informed patient written consent and approval from our local ethics committee (CPP 2015-mai-13909). All the collected placentas resulted from monofetal non-complicated pregnancies, i.e., with no fetal abnormalities, no maternal diseases or treatment (i.e., diabetes, thyroid disorder, hypertension, pre-eclampsia). Placentas included were all macroscopically normal (weight and macroscopic examination).

### 4.2. Isolation and In Vitro Culture of VCT

Chorionic villi were obtained by manual dissection of placental tissues from term placentas as previously described about one hour after delivery [11,55]. Villous tissue was dissected free of membranes, rinsed and minced in Hank’s Balanced Salt Solution (HBSS) 1X. The villous sample was then subjected to sequential enzymatic digestion in HBSS 1X containing 0.2% trypsin (*w/v*), 25 IU/mL DNAse I, 0.1 mM MgSO_4_, 0.1 mM CaCl_2_ and 4% milk (*v/v*). Cell dissociation was monitored by light microscopy. The first three digests were discarded to eliminate residual ST fragments and erythrocytes. Cell suspensions resulting from the following four or five sequential digestions were pooled. Cells were then purified on a discontinuous Percoll gradient (5% to 70% in 14 steps) and their viability was determined in *v/v* solution with trypan blue.

Isolated cells were seeded in DMEM containing 10% Fetal Calf Serum (FCS), 1% Penicillin-Streptomycin and 1% L-Glutamin (complete DMEM) at 1000 cells/mm^2^ during 15 h at 37 °C with 5% CO_2_. After plating, cells were incubated with RTV, LPV or DMSO control during 6 h, 24 h or 48 h (time necessary to allow fusion process). RTV concentrations used ranged from 5 to 20 µM. LPV chosen concentration was 10 µM (=6.288 µg/mL or 6288 ng/mL) corresponding to the mean LPV blood concentration measured in mothers treated for HIV infection. RTV (SML-0491, Sigma-Aldrich^®^ Inc, St-Louis, MO, USA) or LPV (SML-1222, Sigma-Aldrich^®^ Inc, St-Louis, MO, USA) were dissolved in DMSO. Consequently, control DMSO was equivalent to the percentage of DMSO necessary to dissolve LPV.

Cell culture media were collected at different times of culture, supernatants were kept frozen at −80 °C as part of the Equipex 10-PhC/SC-11/243 project “Perinatcollection” until use. Cells were either fixed or snap-frozen for RNA/Protein extraction and stored at −80 °C until use. 

### 4.3. Immunofluorescence Microscopy

Immunocytofluorescence staining was performed after 48 h of incubation with LPV or control DMSO, which corresponds to the time required for ST formation, as previously described [11]. Briefly, cells were fixed and permeabilized in methanol and blocked in Phosphate Buffered Saline (PBS) 1× Bovine Serum Albumine (BSA) 1%-Tween 0.1%. Cells were first incubated overnight at 4 °C with primary polyclonal rabbit antibody to desmoplakin (5 μg/mL, ab16434 abcam^®^, Cambridge, UK). They were then incubated for 1 h at room temperature and protected from light, with secondary goat anti-rabbit antibody conjugated with Alexa Fluor 488 (1/500, A-11008 LifeTechnologies, Carlsbad, CA, USA). Nuclei were stained with 4′,6-diamidino-2-phenylindole (DAPI) and samples were conserved in mounted-medium. Pictures were taken using a BX60 epifluorescence microscope (Olympus) equipped with a 40× oil objective (Olympus 1.00), an ultrahigh-vacuum mercury lamp and a Hamamatsu camera (C4742-95) and analyzed with VisionStage Orca software (v 1.6). The “control immunoglobulin without specific epitope” conditions were used to evaluate the background signal and to set up the acquisition and colorization of pictures. Resulting pictures allowed us to calculate fusion index i.e., (nuclei number in ST-ST number)/total nuclei number. ST was considered when at least two nuclei were not separated by plasmic membrane, observed thanks to desmoplakin staining in green.

### 4.4. Transmission Electronic Microscopy

Trophoblastic cells were seeded for 15 h post isolation and incubated with LPV or control DMSO for 6, 24 or 48 h. Cells were collected after each time by trypsin-EDTA, centrifuged 5 min at 1400× *g*, and washed twice in PB (0.05 M PIPES [piperazine-N,N’-bis(2-ethanesulfonic acid)] buffer, 5 mM CaCl_2_, pH 7.3) for 10 min, then centrifuged 5 min at 1400× *g*. Each sample was fixed during 45 min at room temperature protected from light in PB containing 2.5% glutaraldehyde and 2% paraformaldehyde. After 5 min of centrifugation at 1400× *g*, samples were washed twice for 10 min in PB and then post-fixed first in PB–1% osmium tetroxide (45 min at 4 °C) and then in 1% aqueous uranyl acetate solution for 2 h at room temperature. Samples were then dehydrated in increasing concentrations of ethanol (30%, 50%, 70%, 95% and 100%) followed by ethanol/propylene oxide (1/1 (vol/vol)) and propylene oxide and were finally embedded in Epon epoxy resin. Ultrathin sections (80 nm of thickness) were performed with a Leica ultracut S microtome fitted with a diamond knife (Diatome histoknife Jumbo or Diathome ultrathin). Theses sections were stained with lead citrate and placed on copper grids. The sections were analyzed at 80 kV with a Jeol electron transmission microscope (JEM-100S transmission electron microscope, Croisy sur Seine, France). Acquisitions were made with Gatan software (Gatan Microscopy Suite ^®^, Gatan Inc., AMETEK, Pleasanton, CA, USA).

### 4.5. Hormones Immuno Assays

As previously described [11], total hCG and P4 concentrations were determined in supernatants of culture using ECLIA immuno-assay (Liaison^®^, DiaSorin, Sallugia, Italy). Measuring ranges of both assays were respectively of 1.5–10,000 UI/L and of 0.7–190 nmol/L. Between assay precision expressed by the coefficient of variation (CV %) were <5% and <11% and detection limits were 0.3 UI/L and 0.4 nmol/L respectively.

### 4.6. Western Blots

Total proteins were isolated from cells incubated with RTV, LPV or DMSO using Lysis Buffer (NP40 Cell Lysis Buffer; Invitrogen^TM^, Carlsbad, CA, USA) combined with a protease inhibitor cocktail 100× (1× final; Protease Inhibitor Cocktail Set I, Calbiochem^®^, EMD Chemicals Inc., Merck KGaA, Darmstadt, Germany) and a phosphatase inhibitor cocktail 50× (1× final; Phosphatase Inhibitor Cocktail 50× Set V, Calbiochem^®^, EMD Chemicals Inc., Merck KGaA, Darmstadt, Germany). Proteins (20 µg) were loaded on 4–15% gradient gel (Mini-PROTEAN^®^ TGX^TM^ Precast Gels, BIORAD^®^, Hercules, CA, USA), and were then transferred on nitrocellulose membrane which was blocked with TBS 1×-Milk 5%-Tween 0.1% solution. The resulting proteins blots were probed with anti-MLN64, anti-P450SCC, anti-HSD3B1, anti-OPA1, anti-Mfn2, anti-GRP78, monoclonal mouse anti-actine or anti-vinculine antibodies (references and concentration in Table 1) [11,17]. Actine or vinculine were used as loading control. Addition of secondary goat anti-mouse antibody conjugated with DyLight 680 (1/15,000, #35518 Thermo Fisher Scientific, Waltham, MA, USA) or secondary goat anti-rabbit antibody conjugated with DyLight800 4× PEG (1/15,000, SA5-35571 Thermo Fischer Scientific, Waltham, MA, USA) allowed blots revelation using Odyssey infrared fluorescent system (LI-COR).

### 4.7. Reverse Transcription-Quantitative Polymerase Chain Reaction

Total RNA from human term trophoblast previously transfected with siRNA against IRE1α, ATF6 and PERK were used [17]. Briefly, VCT cells were transfected with 16.6 nM siATF6 (SantaCruz Biotechnology, Labforce, Muttenz, Switzerland), 16,6 nM of siIRE1α (SantaCruz Biotechnology, Labforce, Muttenz, Switzerland) and 16,6 nM of siPERK (SantaCruz Biotechnology, Labforce, Muttenz, Switzerland) or 50 nM control siRNA (SantaCruz Biotechnology, Labforce, Muttenz, Switzerland) using Interferin transfection reagent (Polyplus transfection SA, Illkirch-Graffenstaden, France) and following the manufacturer’s protocol [17]. RNA extracted from trophoblast cells incubated with LPV or DMSO control with or without IRE1α inhibitor (STF-083010) was also analyzed. An amount of 500 ng of total RNA were reversed transcript with SuperScript^®^ III Reverse Transcriptase Kit (Invitrogen^TM^, Carlsbad, CA, USA). The qPCR was performed using cDNA diluted 1/5 in RNase-DNase free water using the the Takyon^TM^ ROX SYBR^®^ MasterMix blue dTTP (Eurogentec, Kaneka, Liège, Belgium). Data were normalized using SDHA, 18S and HPRT as endogenous controls. Used primers are described in Table 2.

### 4.8. Statistical Analysis

Statistical analysis was performed using GraphPad Prism software package^®^ (San Diego, CA, USA). Results were expressed as raw values or mean ± SD. Significant differences (*p* < 0.05) were identified using paired non-parametric student *t* test.

## Figures and Tables

**Table 1:** References and concentrations of primary antibodies.

Target Protein	Concentration	Species	Clonality	Reference
MLN64	1 µg/mL	Rabbit	Polyclonal	ab3478 abcam^®^
P450SCC	1 µg/mL	Rabbit	Polyclonal	ab75497 abcam^®^
HSD3B1	0.18 µg/mL	Rabbit	Monoclonal	ab167417 abcam^®^
Mfn2	1 µg/mL	Mouse	Monoclonal	ab56889 abcam^®^
OPA1	1 µg/mL	Rabbit	Polyclonal	ab42364 abcam^®^
GRP78	1 µg/mL	Rabbit	Polyclonal	G8918 Sigma-Aldrich
Actine	0.2 µg/mL	Mouse	Monoclonal	A5441 Sigma-Aldrich
Vinculine	1 µg/mL	Mouse	Monoclonal	V9131 Sigma-Aldrich

**Table 2:** Sequences of primers used for RT-qPCR.

Target mRNA	Gene Name	Sequences
MLN64	*STARD3*	Forward GAGCGATGGTATCTTGCCGC Reverse CTGCAAAGGATTCTGGGGGT
P450SCC	*CYP11A1*	Forward TTTTTGCCCCTGTTGGATGCA Reverse CCCTGGCGCTCCCCAAAAAT
HSD3B1	*HSD3B1*	Forward AGTACGTCCACTCTTCTGTCCA Reverse TTCTCCTTCACCAAGAGGCG
GRP78	*GRP78*	Forward CGTGGAGATCATCGCCAAC Reverse ACATAGGACGGCGTGATGC
sXBP1	*sXBP1*	Forward CTGAGTCCGAATCAGGTGCAG Reverse ATCCATGGGGAGATGTTCTGG
ATF4	*ATF4*	Forward GTTCTCCAGCGACAAGGCTA Reverse ATCCTGCTTGCTGTTGTTGG
ATF6	*ATF6*	Forward GAGTATTTTGTCCGCCTGCC Reverse CGGGCTAAAAGGTGACTCCA

## Data Availability

Data available on request.

## References

[1] World Health Organization (2016). Consolidated Guidelines on the Use of Antiretroviral Drugs for Treating and Preventing HIV Infection: Recommendations for a Public Health Approach.

[2] Powis K.M., Kitch D., Ogwu A., Hughes M.D., Lockman S., Leidner J., van Widenfelt E., Moffat C., Moyo S., Makhema J. (2011). Increased Risk of Preterm Delivery among HIV-Infected Women Randomized to Protease Versus Nucleoside Reverse Transcriptase Inhibitor-Based HAART During Pregnancy. J. Infect. Dis..

[3] Sibiude J., Warszawski J., Tubiana R., Dollfus C., Faye A., Rouzioux C., Teglas J.-P., Ekoukou D., Blanche S., Mandelbrot L. (2012). Premature Delivery in HIV-Infected Women Starting Protease Inhibitor Therapy During Pregnancy: Role of the Ritonavir Boost?. Clin. Infect. Dis..

[4] Short C.-E.S., Taylor G.P. (2014). Antiretroviral Therapy and Preterm Birth in HIV-Infected Women. Expert Rev. Anti Infect. Ther..

[5] Kariyawasam D., Simon A., Laborde K., Parat S., Souchon P.-F., Frange P., Blanche S., Polak M. (2014). Adrenal Enzyme Impairment in Neonates and Adolescents Treated with Ritonavir and Protease Inhibitors for HIV Exposure or Infection. Horm. Res. Paediatr..

[6] McDonald C.R., Conroy A.L., Gamble J.L., Papp E., Hawkes M., Olwoch P., Natureeba P., Kamya M., Silverman M., Cohan D. (2018). Estradiol Levels Are Altered in Human Immunodeficiency Virus-Infected Pregnant Women Randomized to Efavirenz-Versus Lopinavir/Ritonavir-Based Antiretroviral Therapy. Clin. Infect. Dis..

[7] Rodway M., Zhou F., Benoit J., Yuen B.H., Leung P.C.K. (1988). Differential Effects of 8-Bromo-Cyclic AMP on Human Chorionic Gonadotropin (HCG), Progesterone and Estrogen Production by Term Placental Cells. Life Sci..

[8] Tuckey R.C. (2005). Progesterone Synthesis by the Human Placenta. Placenta.

[9] Lacroix M.C., Guibourdenche J., Frendo J.L., Muller F., Evain-Brion D. (2002). Human Placental Growth Hormone—A Review. Placenta.

[10] Fournier T., Guibourdenche J., Evain-Brion D. (2015). Review: HCGs: Different Sources of Production, Different Glycoforms and Functions. Placenta.

[11] Fraichard C., Bonnet F., Garnier A., Hébert-Schuster M., Bouzerara A., Gerbaud P., Ferecatu I., Fournier T., Hernandez I., Trabado S. (2020). Placental Production of Progestins Is Fully Effective in Villous Cytotrophoblasts and Increases with the Syncytiotrophoblast Formation. Mol. Cell. Endocrinol..

[12] Pasqualini J.R. (2005). Enzymes Involved in the Formation and Transformation of Steroid Hormones in the Fetal and Placental Compartments. J. Steroid Biochem. Mol. Biol..

[13] Pidoux G., Gerbaud P., Tsatsaris V., Marpeau O., Ferreira F., Meduri G., Guibourdenche J., Badet J., Evain-Brion D., Frendo J.-L. (2007). Biochemical Characterization and Modulation of LH/CG-Receptor during Human Trophoblast Differentiation. J. Cell. Physiol..

[14] Pidoux G., Gerbaud P., Dompierre J., Lygren B., Solstad T., Evain-Brion D., Taskén K. (2014). A PKA–Ezrin–Cx43 Signaling Complex Controls Gap Junction Communication and Thereby Trophoblast Cell Fusion. J. Cell Sci..

[15] Gerbaud P., Taskén K., Pidoux G. (2015). Spatiotemporal Regulation of CAMP Signaling Controls the Human Trophoblast Fusion. Front. Pharmacol..

[16] Poidatz D., Dos Santos E., Gronier H., Vialard F., Maury B., De Mazancourt P., Dieudonné M.-N. (2015). Trophoblast Syncytialisation Necessitates Mitochondrial Function through Estrogen-Related Receptor-γ Activation. Mol. Hum. Reprod..

[17] Bastida-Ruiz D., Yart L., Wuillemin C., Ribaux P., Morris N., Epiney M., Martinez de Tejada B., Cohen M. (2019). The Fine-Tuning of Endoplasmic Reticulum Stress Response and Autophagy Activation during Trophoblast Syncytialization. Cell Death Dis..

[18] Walker O.S., Ragos R., Wong M.K., Adam M., Cheung A., Raha S. (2020). Reactive Oxygen Species from Mitochondria Impacts Trophoblast Fusion and the Production of Endocrine Hormones by Syncytiotrophoblasts. PLoS ONE.

[19] Martinez F., Kiriakidou M., Strauss J.F. (1997). Structural and Functional Changes in Mitochondria Associated with Trophoblast Differentiation: Methods to Isolate Enriched Preparations of Syncytiotrophoblast Mitochondria. Endocrinology.

[20] De Los Rios Castillo D., Zarco-Zavala M., Olvera-Sanchez S., Pardo J.P., Juarez O., Martinez F., Mendoza-Hernandez G., García-Trejo J.J., Flores-Herrera O. (2011). Atypical Cristae Morphology of Human Syncytiotrophoblast Mitochondria. J. Biol. Chem..

[21] Meyer J.N., Leuthner T.C., Luz A.L. (2017). Mitochondrial Fusion, Fission, and Mitochondrial Toxicity. Toxicology.

[22] Holland O., Dekker Nitert M., Gallo L.A., Vejzovic M., Fisher J.J., Perkins A.V. (2017). Review: Placental Mitochondrial Function and Structure in Gestational Disorders. Placenta.

[23] Fisher J.J., McKeating D.R., Cuffe J.S., Bianco-Miotto T., Holland O.J., Perkins A.V. (2019). Proteomic Analysis of Placental Mitochondria Following Trophoblast Differentiation. Front. Physiol..

[24] Frank S., Gaume B., Bergmann-Leitner E.S., Leitner W.W., Robert E.G., Catez F., Smith C.L., Youle R.J. (2001). The Role of Dynamin-Related Protein 1, a Mediator of Mitochondrial Fission, in Apoptosis. Dev. Cell.

[25] Rojo M., Legros F., Chateau D., Lombès A. (2002). Characterization of Mammalian Mitofusins. J. Cell Sci..

[26] Chen H., Detmer S.A., Ewald A.J., Griffin E.E., Fraser S.E., Chan D.C. (2003). Mitofusins Mfn1 and Mfn2 Coordinately Regulate Mitochondrial Fusion and Are Essential for Embryonic Development. J. Cell Biol..

[27] Wasilewski M., Semenzato M., Rafelski S.M., Robbins J., Bakardjiev A.I., Scorrano L. (2012). Optic Atrophy 1-Dependent Mitochondrial Remodeling Controls Steroidogenesis in Trophoblasts. Curr. Biol..

[28] Cai H., Chen L., Zhang M., Xiang W., Su P. (2018). Low Expression of MFN2 Is Associated with Early Unexplained Miscarriage by Regulating Autophagy of Trophoblast Cells. Placenta.

[29] Dal Yontem F., Kim S., Ding Z., Grimm E., Ekmekcioglu S., Akcakaya H. (2019). Mitochondrial Dynamic Alterations Regulate Melanoma Cell Progression. J. Cell. Biochem..

[30] Shan A., Li M., Li X., Li Y., Yan M., Xian P., Chang Y., Chen X., Tang N. (2019). BDE-47 Decreases Progesterone Levels in BeWo Cells by Interfering with Mitochondrial Functions and Genes Related to Cholesterol Transport. Chem. Res. Toxicol..

[31] Bielinska M., Boime I. (1995). The Glycoprotein Hormone Family: Structure and Function of the Carbohydrate Chains. New Comprehensive Biochemistry.

[32] Bastida-Ruiz D., Aguilar E., Ditisheim A., Yart L., Cohen M. (2017). Endoplasmic Reticulum Stress Responses in Placentation—A True Balancing Act. Placenta.

[33] Park H.-J., Park S.-J., Koo D.-B., Lee S.-R., Kong I.-K., Ryoo J.-W., Park Y.-I., Chang K.-T., Lee D.-S. (2014). Progesterone Production Is Affected by Unfolded Protein Response (UPR) Signaling during the Luteal Phase in Mice. Life Sci..

[34] Papp E., Mohammadi H., Loutfy M.R., Yudin M.H., Murphy K.E., Walmsley S.L., Shah R., MacGillivray J., Silverman M., Serghides L. (2015). HIV Protease Inhibitor Use during Pregnancy Is Associated with Decreased Progesterone Levels, Suggesting a Potential Mechanism Contributing to Fetal Growth Restriction. J. Infect. Dis..

[35] Wilson R.A., Mesiano S.A. (2020). Progesterone Signaling in Myometrial Cells: Role in Human Pregnancy and Parturition. Curr. Opin. Physiol..

[36] Benirschke K., Burton G.J., Baergen R.N. (2013). Pathology of the Human Placenta.

[37] Handschuh K., Guibourdenche J., Tsatsaris V., Guesnon M., Laurendeau I., Evain-Brion D., Fournier T. (2007). Human Chorionic Gonadotropin Produced by the Invasive Trophoblast but Not the Villous Trophoblast Promotes Cell Invasion and Is Down-Regulated by Peroxisome Proliferator-Activated Receptor-Gamma. Endocrinology.

[38] Bustamante J., Ramírez-Vélez R., Czerniczyniec A., Cicerchia D., Aguilar de Plata A.C., Lores-Arnaiz S. (2014). Oxygen Metabolism in Human Placenta Mitochondria. J. Bioenerg. Biomembr..

[39] Duarte A., Poderoso C., Cooke M., Soria G., Cornejo Maciel F., Gottifredi V., Podestá E.J. (2012). Mitochondrial Fusion Is Essential for Steroid Biosynthesis. PLoS ONE.

[40] Ruiz M., Courilleau D., Jullian J.-C., Fortin D., Ventura-Clapier R., Blondeau J.-P., Garnier A. (2012). A Cardiac-Specific Robotized Cellular Assay Identified Families of Human Ligands as Inducers of PGC-1α Expression and Mitochondrial Biogenesis. PLoS ONE.

[41] Guzel E., Arlier S., Guzeloglu-Kayisli O., Tabak M.S., Ekiz T., Semerci N., Larsen K., Schatz F., Lockwood C.J., Kayisli U.A. (2017). Endoplasmic Reticulum Stress and Homeostasis in Reproductive Physiology and Pathology. Int. J. Mol. Sci..

[42] Burton G.J., Yung H.W., Murray A.J. (2017). Mitochondrial—Endoplasmic Reticulum Interactions in the Trophoblast: Stress and Senescence. Placenta.

[43] Taura M., Kariya R., Kudo E., Goto H., Iwawaki T., Amano M., Suico M.A., Kai H., Mitsuya H., Okada S. (2013). Comparative Analysis of ER Stress Response into HIV Protease Inhibitors: Lopinavir but Not Darunavir Induces Potent ER Stress Response via ROS/JNK Pathway. Free Radic. Biol. Med..

[44] Yeh R.F., Gaver V.E., Patterson K.B., Rezk N.L., Baxter-Meheux F., Blake M.J., Eron J.J.J., Klein C.E., Rublein J.C., Kashuba A.D.M. (2006). Lopinavir/Ritonavir Induces the Hepatic Activity of Cytochrome P450 Enzymes CYP2C9, CYP2C19, and CYP1A2 But Inhibits the Hepatic and Intestinal Activity of CYP3A as Measured by a Phenotyping Drug Cocktail in Healthy Volunteers. JAIDS J. Acquir. Immune Defic. Syndr..

[45] Besse A., Stolze S.C., Rasche L., Weinhold N., Morgan G.J., Kraus M., Bader J., Overkleeft H.S., Besse L., Driessen C. (2018). Carfilzomib Resistance Due to ABCB1/MDR1 Overexpression Is Overcome by Nelfinavir and Lopinavir in Multiple Myeloma. Leukemia.

[46] Papp E., Balogun K., Banko N., Mohammadi H., Loutfy M., Yudin M.H., Shah R., MacGillivray J., Murphy K.E., Walmsley S.L. (2016). Low Prolactin and High 20-α-Hydroxysteroid Dehydrogenase Levels Contribute to Lower Progesterone Levels in HIV-Infected Pregnant Women Exposed to Protease Inhibitor–Based Combination Antiretroviral Therapy. J. Infect. Dis..

[47] Hong-Brown L.Q., Brown C.R., Huber D.S., Lang C.H. (2008). Lopinavir Impairs Protein Synthesis and Induces EEF2 Phosphorylation via the Activation of AMP-Activated Protein Kinase. J. Cell. Biochem..

[48] Yung Y., Maman E., Ophir L., Rubinstein N., Barzilay E., Yerushalmi G.M., Hourvitz A. (2014). Progesterone Antagonist, RU486, Represses LHCGR Expression and LH/HCG Signaling in Cultured Luteinized Human Mural Granulosa Cells. Gynecol. Endocrinol..

[49] Guitart-Mampel M., Hernandez A.S., Moren C., Catalan-Garcia M., Tobias E., Gonzalez-Casacuberta I., Juarez-Flores D.L., Gatell J.M., Cardellach F., Milisenda J.C. (2017). Imbalance in Mitochondrial Dynamics and Apoptosis in Pregnancies among HIV-Infected Women on HAART with Obstetric Complications. J. Antimicrob. Chemother..

[50] Ganta K.K., Chaubey B. (2019). Endoplasmic Reticulum Stress Leads to Mitochondria-Mediated Apoptosis in Cells Treated with Anti-HIV Protease Inhibitor Ritonavir. Cell Biol. Toxicol..

[51] Chen L., Jarujaron S., Wu X., Sun L., Zha W., Liang G., Gurley E.C., Studer E.J., Hylemon P.B., Pandak W.M. (2009). HIV Protease Inhibitor Lopinavir-Induced TNF-a and IL-6 Expression Is Coupled to the Unfolded Protein Response and ERK Signaling Pathways in Macrophages. Biochem. Pharmacol..

[52] Zha B.S., Wan X., Zhang X., Zha W., Zhou J., Wabitsch M., Wang G., Lyall V., Hylemon P.B., Zhou H. (2013). HIV Protease Inhibitors Disrupt Lipid Metabolism by Activating Endoplasmic Reticulum Stress and Inhibiting Autophagy Activity in Adipocytes. PLoS ONE.

[53] Brüning A., Kimmich T., Brem G.J., Buchholtz M.L., Mylonas I., Kost B., Weizsäcker K., Gingelmaier A. (2014). Analysis of Endoplasmic Reticulum Stress in Placentas of HIV-Infected Women Treated with Protease Inhibitors. Reprod. Toxicol..

[54] Gallagher C.M., Garri C., Cain E.L., Ang K.K.-H., Wilson C.G., Chen S., Hearn B.R., Jaishankar P., Aranda-Diaz A., Arkin M.R. (2016). Ceapins Are a New Class of Unfolded Protein Response Inhibitors, Selectively Targeting the ATF6α Branch. eLife.

[55] Alsat E., Haziza J., Evain-Brion D. (1993). Increase in Epidermal Growth Factor Receptor and Its Messenger Ribonucleic Acid Levels with Differentiation of Human Trophoblast Cells in Culture. J. Cell. Physiol..

